# Clinical, laboratory, and chest radiographic characteristics of COVID-19 associated severe pediatric pneumonia. *A retrospective study*

**DOI:** 10.15537/smj.2022.43.12.20220420

**Published:** 2022-12

**Authors:** Ali A. Asseri, Saleh M. Al-Qahtani, Mohammed A. Algathradi, Ibrahim A. Alzaydani, Ahmed A. Al-Jarie, Ibrahim A. Al-Benhassan, Ibrahim A. AlHelali, Mona S. Alassiri, Ali A. Alrmelawi, Abdelwahid S. Ali

**Affiliations:** *From the Department of Child Health (Asseri, Al-Qahtani); from the Department of Radiology (Algathradi); from the Department of Microbiology and Clinical Parasitology (Ali), College of Medicine, King Khalid University, from the Department of Pediatrics (Alzaydani, Al-Jarie, Alassiri); from the Department of Pediatric Critical Care Unit (Al-Benhassan, AlHelali); and from the Department of Microbiology (Alrmelawi), Abha Maternity and Children Hospital, Abha, Kingdom of Saudi Arabia.*

**Keywords:** children, coronavirus disease-19, COVID-19, SARS-CoV-2, pneumonia

## Abstract

**Objectives:**

To evaluate the demographics, clinical presentation, laboratory data, chest radiographs, and outcomes of pediatric patients with critical coronavirus disease 2019 (COVID-19).

**Methods:**

This retrospective study included 34 children who were diagnosed with severe COVID-19 pneumonia between August 2020 and July 2021. Severe pneumonia was defined as fever, respiratory distress (tachypnea, chest retractions, and hypoxia [oxygen saturation <90% in room air]), and obvious infiltrations on chest radiography.

**Results:**

Ages of the patients ranged from newborns to 12 years old, with a median of 24 months (interquartile range: 12-72 months). Preschool-aged children were the most common age group (44%). Levels of inflammatory markers (C-reactive protein, ferritin, and procalcitonin) were elevated in most patients. A total of 13 patients developed severe acute respiratory distress syndrome (ARDS), while 4 developed multiorgan failure. Despite receiving supportive therapy, 2 (5.9%) patients died due to severe septic shock and multiorgan failure. One deceased patient was born prematurely at 30 weeks, while the other had chronic granulomatous disease.

**Conclusion:**

This study described a single-center cohort of pediatric patients with severe COVID-19 pneumonia. In this cohort, children with cardiopulmonary comorbidities and ARDS had a high mortality and long-term morbidity, as observed in other pediatric studies.

**C**oronavirus disease 2019 (COVID-19) is a viral respiratory infection caused by the emerging novel coronavirus, severe acute respiratory syndrome coronavirus 2 (SARS-CoV-2).^1^ This virus was first reported in Wuhan, the capital city of Hubei province, China, in late December 2019, with speculation of an animal source.^2^ Genomic and biological studies have confirmed that the virus belongs to the genus *Betacoronavirus* of the family *Coronaviridae*.^3^ The clinical presentation of COVID-19 includes fever, dry cough, dyspnea, myalgia, fatigue, and pneumonia, with some variations among infected individuals.^4^ Other symptoms, including dizziness, generalized weakness, vomiting, and diarrhea were also observed.^5^ Deaths among COVID-19 patients were observed to be correlated with acute respiratory distress syndrome (ARDS).^6^ Although the virus is reportedly highly pathogenic in all age groups, several clinical and epidemiological studies have indicated that disease severity and fatalities are higher among adults, particularly the elderly and lower among children.^7,8^ Additionally, immunosuppressed individuals and those suffering from chronic diseases, such as diabetes mellitus, hypertension, and cardiovascular and pulmonary diseases are more prone to severe and fatal COVID-19 infections.^8^

During the early days of the COVID-19 pandemic, several reports indicated a mild course and benign viral infection among pediatric patients.^9^ Subsequently, with the progression of studies pertaining to the disease pathology, symptomatology, and clinical phenotypes among pediatric patients, increasingly severe and critical pediatric COVID-19 cases have been reported.^8,10^ These severe cases are associated with pre-existing diseases and concurrent multisystem inflammatory syndrome in children.^10-12^ In the present study, we evaluated the demographic characteristics, clinical presentation, laboratory data, chest radiographs (CXR), and outcomes of pediatric patients with severe COVID-19-associated pneumonia.

## Methods

This single-center retrospective observational study included 34 children diagnosed with severe laboratory-confirmed COVID-19 pneumonia who were hospitalized in the Pediatric Intensive Care Unit (PICU) of Abha Maternity and Children Hospital, Abha, Saudi Arabia, between August 2020 and July 2021. We enrolled patients ranging from newborns to 12 years old with confirmed COVID-19 severe pneumonia through a nasopharyngeal real-time polymerase chain reaction test. Patients with fever, respiratory distress (tachypnea, chest retractions, and hypoxia [oxygen saturation {SpO2} <90% in room air]), and obvious infiltrations on CXR were diagnosed with severe pneumonia. We included patients who required PICU admission, owing to severe respiratory distress and oxygen supplementation of >5 L/minute. We excluded patients with mild COVID-19 pneumonia who required general pediatric ward care.

This study was carried out in accordance with the guidelines of the Declaration of Helsinki and was approved by the Ethics Committee of King Khalid University, Ahba, Saudi Arabia, on October 16, 2021 (approval number: E.C.M.# 2021-5818) - (HAPO-06-B-001). Additionally, the requirement for informed consent was waived, owing to the observational nature of the study.

Medical charts of the enrolled patients were reviewed. Demographic data, including age, gender, clinical symptoms, and comorbidities, were analyzed. Initial laboratory data were collected, including white blood cell and differential counts, platelet count, hemoglobin level, complete metabolic panel, and inflammatory marker levels. A pediatric radiologist evaluated the CXR and computed tomography (CT) findings. The findings were described based on the following features: abnormal opacities, distribution of abnormal opacities, and unilateral or bilateral abnormal opacities. The CXR and CT patterns were retrospectively evaluated for the presence, distribution, and characteristics of ground-glass opacities and consolidation. The clinical courses included the total number of PICU hospitalizations, number of days of high-flow nasal cannula (HFNC) use, number of days of mechanical ventilator (MV) use, and survival outcome.

### Statistical analysis

Data were collected, coded, and entered into the Statistical Package for the Social Sciences, version 22.0 (IBM Corp., Armonk, NY, USA). A descriptive analysis based on the frequency and percentage distribution was carried out for all variables, including demographic data, symptoms, medications, and comorbidities. Additionally, the mean and standard deviation (SD) was calculated for normally distributed quantitative variables, whereas the range and median with interquartile range (IQR) were calculated for skewed numerical variables.

## Results

The demographic and clinical characteristics, including the initial vital signs in the emergency room are summarized in Table 1. The age of the patients ranged between 0-12 years, with a median of 24 months (IQR: 12-72 months). This study included 16 male and 18 female patients, of whom 20 (59%) had gastrointestinal manifestations. All patients had significant hypoxia upon presentation to the emergency room (median room air oxygen saturation: 75%, IQR: 70-82%). A total of 12 patients required immediate invasive ventilation in the emergency room. Septic shock occurred in 2 patients. The prevalence of comorbid disorders included cardiopulmonary disorders, primary immunodeficiency, and neuromuscular disorders in 31 of 34 (91.2%) patients. Median heart rate of the enrolled patients was 147.5 beats per minute, and most patients required a high oxygen concentration level with the introduction of HFNC or MV. Median fraction of inspired oxygen was 100, with an IQR of 80-100.

**Table 1:** - Baseline characteristics of the patients with severe COVID-19 pneumonia (N=34).

Variables	n (%)
Age in months, median (IQR)	24 (12-72)
***Age categories***	
Infants	11 (32.4)
Preschool age	15 (44.0)
School age	8 (23.5)
Gender (male)	16 (47.0)
***Symptoms and signs***	
GI symptoms (vomiting/diarrhea)	20 (59.0)
SpO2 on room air, median (IQR)	75 (70-82)
Respiratory failure requiring mechanical ventilation	12 (35.0)
Signs of shock or multi-organ failure	2 (5.9)
Comorbidities	31 (91.2)
***Vital signs at admission, median (IQR)***	
Heart rate	147.5 (120-181)
FiO_2_	100 (80-100)

Values are presented as a number and percentage (%) or as median and interquartile range (IQR). GI: gastrointestinal, SpO2: peripheral oxygen saturation, FiO2: the fraction of inspired oxygen

**Figure 1:**
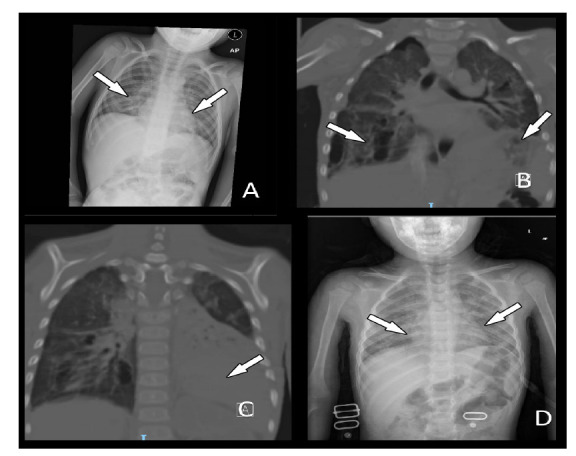
- Progression of chest radiography (CXR) and computed tomography (CT) findings in a pediatric COVID-19 patient. A) CXR of the patient on admission showed bilateral mid-lung zone opacifications. B&C) During admission to the pediatric intensive care unit, coronal CT images of the same patient showed multiple right sided pneumatoceles, bilateral ground-glass opacification, and left lower lobe consolidation. D) CXR of the patient at the 3-month post-discharge follow-up showed recovering bilateral ground-glass opacifications.

**Figure 2:**
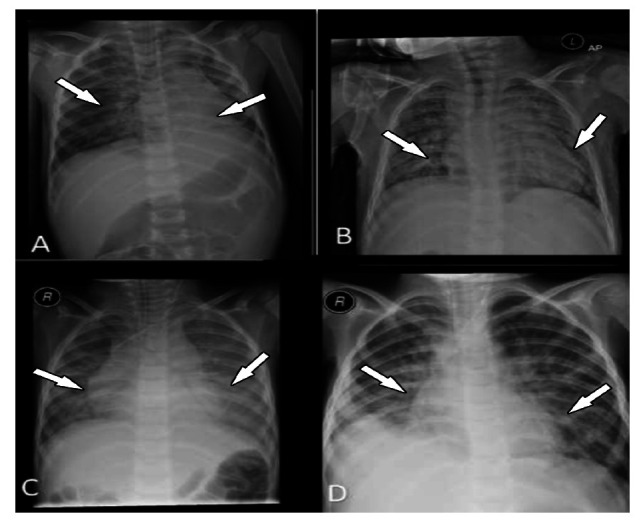
- Chest radiographs of 4 pediatric patients with positive COVID-19 results. A) A 3-year-old male patient with COVID-19; initial chest radiography after intubation shows bilateral central ground-glass opacities with left lower lobe consolidation. B) An 8-year-old male patient with underlying severe bronchopulmonary dysplasia was admitted with COVID-19; chest radiography showed bilateral ground-glass opacities (more on the left). C) Chest radiography of a 4-year-old patient showing bilateral ground-glass opacity. D) Chest radiography of a 5-year-old showing bilateral consolidation with right lower lobe localization. This patient eventually died.

The patients’ initial laboratory data are presented in Table 2. Inflammatory markers (C-reactive protein [CRP], ferritin, erythrocyte sedimentation rate [ESR], and procalcitonin) were elevated in most patients. Four (11.8%) patients had acute kidney injury (AKI) that was treated conservatively and did not require dialysis. Chest radiography was carried out in 34 patients and CT was carried out in 18 patients. The CXR showed peribronchial thickening in 30 (88.2%) patients, consolidation in 26 (76.5%), and ground-glass opacities (GGO) in 23 (67.6%). Additionally, bilateral lung opacifications were seen in 22 (67.7%) patients, and the lower lobes were the most commonly involved areas in 29 (85.3%) patients. An example of the progression of radiological findings on CXRs and chest CT in one patient is shown in Figure 1. Chest radiography findings of some of the patients are shown in Figure 2. The most common findings detected on chest CT were GGO in 17 (94.4%) patients and parenchymal infiltration in 16 (88.9%). Additionally, 4 (11.8%) patients had diffuse air leak syndrome (pneumothorax, pneumomediastinum, and subcutaneous emphysema) that occurred after MV.

**Table 2:** - Baseline laboratory findings of the pediatric patients.

Blood and biochemical tests	n	Children’s reference range	Values
White blood cells (×10^9^/L)	34	4500-11000	9.21 (6-12.9)
Absolute lymphocyte count (per mm^3^)	31	1000-4800	2340 (1540-4610)
Absolute neutrophil count (per mm^3^)	31	1800-7700	4740 (2470-10300)
Hemoglobin (g/dL)	34	12.0-16.0	12 (10.5-13)
Platelets (×10^9^/L)	34	150,000-450,000	281 (186-334)
CRP (mg/dL)	22	<0.30	5 (0.5-14.5)
ESR (mm/hr)	29	0-13	32 (15-55)
Ferritin, µg/l	8	30-300	351 (144-1293)
Procalcitonin, µg/l	13	0.00-0.08	0.34 (0.04-5.5)
Troponin, ng/ml	12	<2.0	3.8 (1.5-46)
D-dimer (µg/L)	14	<500	970 (725-3003)
BUN (mg/dL)	31	8.0-25	13 (8-19)
Creatinine (mg/dL)	31	0.30-1.00	0.4 (0.3-0.5)
Sodium (mEq/L)	31	135-145	137 (134-139)
ALT (IU/L)	29	10-55	29 (18-43.5)
AST (IU/L)	29	9.0-32	29 (22-53.5)
Albumin (g/dL)	26	3.4-5.4	3.1 (2.8-3.7)
Total bilirubin (mg/dL)	19	0.0-1.0	0.5 (0.3-1)
LDH (IU/L)	17	13-60	472 (310.5-716)

Values are presented as median and interquartile range (IQR). CRP: C-reactive protein, ESR: erythrocyte sedimentation rate, BUN: blood urea nitrogen, ALT: alanine transaminase, AST: aspartate transaminase, LDH: lactate dehydrogenase

Tables 3 and 4 show the therapies, outcome measures, and complications of the study groups. A total of 12 patients had septic shock and required immediate endotracheal intubation in the emergency room, and 7 of the 12 required inotropic support. Hydroxychloroquine was administered to 9 (26.5%) patients. Systemic steroids (methylprednisolone and oral prednisolone) was administered to 58.8% of the patients and favipiravir was administered to 94.1% of the patients. Of the 34 patients, 12 (35.3%) who were severely ill and required invasive ventilation received intravenous immunoglobulin (manufactured by Instituto Grifols S.A., Barcelona, Spain). All patients received 2 or more broad-spectrum antibiotics (vancomycin, ceftriaxone, meropenem, and piperacillin/tazobactam). High-flow nasal cannula was the most commonly used respiratory support modality in 31 of the 34 (91.2%) patients. Six patients required prolonged HFNC use after MV weaning and were eventually weaned off HFNC. The median hospital stay was 10 days (range: 3-105 days)and the median PICU stay was 7 days (range: 1-91 days).

**Table 3:** - Interventions and therapeutics options.

Variables	Values
***Medical treatment***	
Inotropes	7 (20.6)
Systemic steroid	20 (58.8)
IVIG	12 (35.3)
Aspirin	3 (8.8)
Azithromycin	34 (100)
≥2 antibiotics	34 (100)
Favipiravin	32 (94.1)
Hydroxychloroquine	9 (26.5)
***Respiratory support***	
HFNC	31 (91.2)
MV	11 (32.4)
Days on HFNC	3 (1-20)
Days on MV	10 (1-60)
Days on PICU	7 (1-91)
Total oxygen days during the hospitalization	7 (3-80)
The total days of hospital stay	10 (3-105)

Values are presented as a number and precentage (%) or as median and interquartile range (IQR). IVIG: intravenous immunoglobulin, HFNC: high-flow nasal cannula therapy, MV: mechanical ventilation, PICU: pediatric intensive care unit

**Table 4:** - Complications and the outcomes.

Variables	Values
***Complications***	
Multiorgan failure	4 (11.8)
Seizures	5 (14.8)
ARDS	13 (38.2)
Air leak	4 (11.8)
***Outcomes at the time of discharge***	
Tracheostomized	3 (8.8)
Oxygen dependent	6 (17.6)
Discharged alive	32 (94.0)
A 3-months follow-up	26 (76.5)

Values are presented as a number and precentage (%).

ARDS: acute respiratory distress syndrome

A total of 13 patients developed severe ARDS, while 4 developed multiorgan failure. Two (5.9%) patients died of severe septic shock and multiorgan failure despite receiving all suppurative therapies, including MV, vasopressors, intravenous immunoglobulin (IVIG), methylprednisolone, favipiravir, and broad-spectrum antibiotics. One deceased patient was born premature at 30 weeks. Another patient had primary immunodeficiency disease (autosomal recessive chronic granulomatous disease type 1 with a homozygous pathogenic variant identified in the NCF1 gene). Three patients required tracheostomy insertion due to failure of extubation trials; thus, they were discharged with tracheostomy and oxygen supplementation. At 3 months post-hospitalization, 26 (76.5%) patients were re-evaluated. Only 3 patients were on home oxygen, and 2 of the 3 patients who required tracheostomy insertion were decannulated, of which they carried out well without any chronic respiratory problems (cough, dyspnea, and stridor). At the time of writing this report, one patient had a tracheostomy in place for severe subglottic stenosis due to prolonged mechanical ventilation.

## Discussion

Severe acute respiratory syndrome coronavirus 2 is presumed to be less virulent and more severe in children than in adults.^7-10,13^ Thus, most published studies have focused on the clinical and radiographic characterization of COVID-19 pneumonia in adults.^1,2,6,7^ However, recent case reports and case series have demonstrated that SARS-CoV-2 can cause severe infections and invasive complications in children.^8,10,14^ This study describes the clinical symptoms, laboratory data, chest radiographs, and outcomes of critically ill children with COVID-19 pneumonia. Despite the low prevalence of severe COVID-19 pneumonia in pediatric patients, children with cardiopulmonary and immunodeficiency disorders are at a high risk of morbidity and mortality.^8,13,14^ In our cohort, the mortality rate was 5.9%; 2 of the 34 patients died due to severe ARDS and multiorgan failure. One patient had underlying chronic granulomatous disease type 1 and developed severe bacterial pneumonia during the PICU stay. The other patient had moderate bronchopulmonary dysplasia and required prolonged neonatal ICU admission immediately after birth for 2 months. He was readmitted at 3 months with severe COVID-19 pneumonia and died due to severe ARDS. The reported mortality of pediatric COVID-19 pneumonia ranges between <1-5%, particularly in children with underlying comorbidities.^13^

The epidemiological and clinical characteristics of the children enrolled in our study were similar to those previously described.^8,10,11,13^ In the current cohort, most of the children were infants and preschoolers. In addition, the majority of the enrolled children had preexisting comorbidities. The most prevalent symptoms were fever, cough, dyspnea, and hypoxia. A total of 12 patients required immediate invasive ventilation upon arrival at the emergency room and all had comorbid conditions. It is now clear that children with chronic diseases are more susceptible to severe COVID-19 manifestations.^10-13^ Despite progression to severe ARDS, specifically in the presence of comorbidities, recovery is faster, probably due to a better immune response and reduced expression and maturity of angiotensin-converting enzyme 2.^13^ Angiotensin-converting enzyme 2 is a transmembrane receptor found on the apical membranes of respiratory epithelial cells, mainly in type II pneumocytes.^13^

Most of the children had normal laboratory results; however, elevated inflammatory parameters were observed. In our study, 77% (17 of 22) of the patients had a CRP level of more than 0.3 mg/dL; the highest recorded value was 14.5 mg/dL. The median ESR was 32 mm/h, with the highest recorded value of 55 mm/h. Other inflammatory markers showed similar elevation trend (ferritin, procalcitonin, and D-dimer). Several authors have reported elevated inflammatory markers in children with SARS-CoV-2 infection, with concurrent worsening of respiratory status, progression to severe ARDS, and multiorgan dysfunction.^8,10,13^ Recent research has shown that children with severe COVID-19 pneumonia have significantly higher levels of inflammatory markers.^13^

Acute kidney injury has been reported in as many as 29% of children with severe COVID-19 disease.^15^ Kari et al^15^ reported AKI in 21% of a cohort of 89 COVID-19-positive children requiring hospital admission, specifically PICU admission. The exact etiology of AKI-related COVID-19 infection in children is unknown and requires further pathobiological studies. The proposed mechanisms for AKI in COVID-19-infected children include direct viral injury, dysregulated inflammation with cytokine storm, vascular injury, hypovolemia, heart failure, sepsis, and dehydration.^15^ However, the cause of AKI is complex and involves multiple mechanisms.

Despite the lack of standardized pharmacological therapies for severe pediatric COVID-19 pneumonia, several immunomodulatory therapies and antiviral agents have been used to treat complications associated with SARS-CoV-2 infection.^8,12,14^ Systemic steroids was used in 58.9% of the patients and IVIG was used in 35.3% of the patients. Steroids were administered to patients with ARDS, whereas IVIG was administered to patients with severe septic shock. Favipiravir has not been approved for COVID-19 treatment, owing to its lack of proven efficacy; however, it has been used in several countries in patients with severe COVID-19.^12,14^ In our cohort, favipiravir was administered to 94.1% of the patients. Broad-spectrum antibiotics were administered to all patients, with ceftriaxone and vancomycin being the most commonly used antibiotics. Overall, 26% of our patients received hydroxychloroquine, which has a very good safety profile.

Chest CT is an important diagnostic tool for adult patients with COVID-19 pneumonia.^1,6^ Ground-glass opacities in the middle and peripheral lung zones are common radiological abnormalities.^1,6^ Ground-glass opacities is a hazy opacity that does not obscure underlying bronchovascular markings. It identifies the existence of fluid (transudate or exudate) partially filling the air spaces in the lungs along with interstitial thickening or partial alveolar collapse (6). Similar to other studies, the most prominent CT findings in the current study were GGOs (94.4%) and parenchymal infiltration (88.9%). Four (11.8%) patients developed pneumothorax after MV.

The findings of the current study have public health implications, thereby highlighting the importance of vaccinating children with cardiopulmonary disorders to prevent COVID-19 sequelae and public transmission of COVID-19.

### Study limitations

Its retrospective design, small sample size from a single center, and lack of a control group, which limit the generalizability of the findings to other pediatric populations. Additionally, the lack of trends in inflammatory marker results affected the radiological and laboratory phenotyping of pediatric ARDS in our study.

In conclusion, this study describes a single-center cohort of pediatric patients with critical COVID-19 pneumonia requiring ICU admission with detailed clinical and radiological outcomes. Similar to other pediatric studies, children with cardiopulmonary comorbidities and ARDS have high mortality and long-term morbidities; however, our small sample size prevented definitive conclusions. A further prospective large study is certainly required to study the risk factors of severe pediatric COVID-19 pneumonia.
